# Professor Tiedemann on the Glands of Duverney, Bartholinus, or Cowper in the Human Female; and on Obliquity in the Form and Position of the Uterus

**Published:** 1843-07

**Authors:** 


### ART. IX.

*Friederich Tiedemann, Professor in Heidelberg, von den Duverneyschen Bartholinschen, oder Cowperschen Drüsen des Weibs und der schiefen Gestaltung und Lage der Gebärmutter.—Heidelberg,* 1840. 4to, pp.42.

*Professor Tiedemann on the Glands of Duverney, Bartholinus, or Cowper in the Human Female; and on Obliquity in the form and position of the Uterus.* Four Plates.—*Heidelberg and Leipsic,* 1840.

I. ABOUT the middle of the seventeenth century J. C. Duverney discovered (at either side of the entrance of the vagina in the cow) a duct leading to a large lobulated gland which was covered by the skin and the constrictor vaginæ muscle. Bartholinus, to whom Duverney mentioned the existence of these organs, discovered them in the human female, and compared them to the prostate in the male. Duverney confirmed the observation of Bartholinus, and after him Peyer, Harder, Morgagni, Santorini, and others, saw and described them. Haller did not succeed in detecting them, but denied their existence, and asserted that the supposed glands to which the ducts of Bartholinus led were mere mucous crypts. Haller's denial weighed more than the assertions of anatomists for the previous half century, and it was soon forgotten that any such structures had ever been observed. After more than a century they were rediscovered by Mr. Taylor, who described them in the Dublin Journal, vol. xiii., and they are briefly alluded to in Mr. Guthrie's work on Diseases of the Bladder.

The attention of Professor Tiedemann was called to these organs by a visit which he paid to Hamburg in 1830, when Dr. Fricke pointed out to him in the venereal hospital, the liability of the follicles at the entrance of the vagina to inflammation and ulceration. On referring to the writings of various anatomists, he was surprised to find in some a minute description of the form and situation of two large glands placed at the entrance of the vagina, and accordingly he proceeded to examine them for himself. After a little dissection he discovered them.

"These glands are situated at each side of the entrance of the vagina, beneath the skin covering the posterior or inferior part of the labia. They are likewise covered by the superficial fascia of the perineum, and by the fibres of the constrictor vaginæ. They here occupy a space included between the lower end of the vagina and the ascending ramus of the ischium, and the ramus of the clitoris and erector clitoridis muscle. Above them lie those fibres of the levator ani which take their origin from the ischium, and behind them are the transversi perinæi muscles. The glands are enveloped in very loose cellular tissue, the removal of which requires very careful dissection in order not to cut away the glands with it. They are rounded but elongated, flat and bean-shaped. Their long diameter varies from 5 to 10 lines: their transverse diameter from 2½ to 4¼, and their thickness from 2¼ to 3 lines." (p. 13.)

Like Cowper's glands in the male they are not invariably present. They frequently differ in size on the two sides, and sometimes the gland on one side is absent. In advanced age they diminish in size, and even totally disappear.

From the anterior edge of the upper part of each gland an excretory duct runs horizontally forwards and inwards, beneath the constrictor vaginæ, to the inner side of the nympha, where its opening may be detected just in front of the carunculæ mystiformes, and surrounded by several small mucous follicles. The secretion of these organs is a thick, tenacious, grayish-white fluid, which is emitted in large quantities during sexual intercourse, and is doubtless the matter erroneously supposed by Hippocrates and the medical writers of antiquity to be the female semen. The uses which this secretion serves are unknown. It is scarcely likely that it answers merely the mechanical purpose of lubricating the parts during copulation. Probably its admixture with the semen answers some important purpose connected with impregnation. Perhaps it is a vehicle for the fecundating principle of the semen, an office which appears to be in part fulfilled by the secretion from the prostate, and Cowper's glands in the male. The existence of these bodies in the females of all animals, in which the males are furnished with Cowper's glands, is another strong argument in favour of the analogy of their functions.

These glands are subject to various morbid affections, and are especially liable to become diseased in the progress of virulent gonorrhœas. Tiedemann found the excretory ducts obliterated in the body of a woman who had suffered from inflammation of the mucous membrane of the vagina, while the lobules of the glands were dilated to little cells, and contained a reddish tenacious fluid. In the body of a young woman who had died of puerperal fever these organs were red, inflamed, and much swollen.

II. The subject of the second essay was briefly referred to in our review of Naegele's work on Obliquity of the Pelvis. Tiedemann, as might be expected, treats the theme as an anatomist, not as an obstetrician, though he glances at the influence of this malformation of the uterus upon labour. In the course of forty years Professor Tiedemann has met eight times with obliquity in the form of the uterus. The degree of the malformation differed much in the different cases, and it is not very easy to convey a just notion of it to the reader without the aid of plates. In one instance two thirds of the uterus lay to the right of a perpendicular line drawn from the centre of the fundus. With this deformity of the uterus there was usually combined an inequality in the length of the broad and raised ligaments. In one instance the left side of the fundus at the point where the Fallopian tube enters formed a very acute angle, and stood somewhat higher than the right side, facts which clearly point to the mode in which this deformity originates, and show it to be congenital. One of the eight observations, too, relates to an infant which died immediately after birth, and proves that this state is an original malformation, and not a condition acquired in after life. In the seven other instances the subjects were adult, but none of them showed any sign of ever having been pregnant, though in five the hymen was destroyed. This circumstance might lead to the supposition that obliquity of the uterus occasions sterility, perhaps from the curved and narrow state of the canal of the cervix. There is an instance to the contrary however in Cranmerarius and Pfister, *Specimen experimentorum circa generationem hominis et animalium*, p. 10. The same case likewise illustrates an assertion of some of the older writers on midwifery, (concerning which much doubt has been expressed), that obliquity of the uterus renders parturition extremely hazardous and difficult, the woman having died undelivered.

It is evident from the above facts, that an oblique uterus retains this form during labour, and thus Boer's ingenious but hypothetical distinction between obliquity in the form and in the situation of the uterus is fully confirmed.

The observations of obliquity in the form of the uterus before the appearance of Tiedemann's paper are very few; obliquity in its situation, from unequal length of its ligaments, has been noticed by many anatomists, though by but few midwifery writers. The observations of obliquity from this cause related by Tiedemann, or quoted by him from others, do not contain anything novel.

One question of some interest still remains; namely, the circumstances which may produce obliquity of the uterus after birth. In two cases of lateral curvature, with the convexity towards the right side, Morgagni found the uterus inclined to the right. In any form of lateral curvature too the uterus is likely, in the event of pregnancy occurring, to assume a position more or less oblique. Lameness when one leg is shorter than the other may be associated with it. It has also been found associated with obliquity of the pelvis. There are likewise many causes, such as adhesions from peritonitis, or tumours of various kinds, which may throw the uterus out of its natural position; but there is no foundation for the notion, first propounded by Gradi, that a lateral insertion of the placenta may produce obliquity of the uterus.

An appendix contains four observations by Professor Stotz, of Strasburg, of curvature of obliquity in the form of the uterus.

We cannot close this abstract of these two tracts without expressing the wish that there were more in our own country disposed to imitate Professor Tiedemann, who, though full of years and honours, continues with unabated zeal the pursuits of his youth.

